# Assembly of the Cutin Polyester: From Cells to Extracellular Cell Walls

**DOI:** 10.3390/plants6040057

**Published:** 2017-11-18

**Authors:** Bénédicte Bakan, Didier Marion

**Affiliations:** INRA, Biopolymers Interactions Assemblies Research unit, La Géraudière, 44316 Nantes Cedex 3, France; didier.marion@inra.fr

**Keywords:** cutin, polysaccharides, polyester, cutin synthase, GDSL-lipase

## Abstract

Cuticular matrices covering aerial plant organs or delimiting compartments in these organs are composed of an insoluble hydrophobic polymer of high molecular mass, i.e., cutin, that encompass some cell wall polysaccharides and is filled by waxes. Cutin is a polyester of hydroxy and-or epoxy fatty acids including a low amount of glycerol. Screening of *Arabidopsis* and more recently of tomato (*Solanum lycopersicum*) mutants allowed the delineation of the metabolic pathway involved in the formation of cutin monomers, as well as their translocation in the apoplast. Furthermore, these studies identified an extracellular enzyme involved in the polymerization of these monomers, i.e., cutin synthase 1 (CUS1), an acyl transferase of the GDSL lipase protein family. By comparing the structure of tomato fruit cutins from wild type and down-regulated CUS1 mutants, as well as with the CUS1-catalyzed formation of oligomers in vitro, hypothetical models can be elaborated on the polymerization of cutins. The polymorphism of the GDSL-lipase family raises a number of questions concerning the function of the different isoforms in relation with the formation of a composite material, the cuticle, containing entangled hydrophilic and hydrophobic polymers, i.e., polysaccharides and cutin, and plasticizers, i.e., waxes.

## 1. Introduction

Cuticular layers are ubiquitous protective barriers of plants that cover their aerial organs or separate their internal tissues. Cuticular layers are composed of an insoluble hydrophobic polymer named cutin, a polyester of hydroxy and-or epoxy fatty acids, that are covered and filled by waxes, a complex mixture of derivatives of very long-chain fatty acids (alkanes, alcohols, esters) and terpenoids. The chemical diversity and complexity of surface lipids has been described in many plants and plant organs since the pioneering works performed in the sixties [1,2]. Cutin and wax deposition occurs on a cell wall polysaccharide matrix. Polysaccharides are therefore a significant polymer component of cuticular layers. Indeed, in most plant surfaces, a gradient is formed where the external surface, i.e., the cuticle proper, is composed of cutin and waxes while the internal part, i.e., the cuticle layer, contains cutin, waxes and polysaccharides [3]. This chemical gradient was already described in the mid nineteenth century [4]. Such a complex supramolecular assembly gives unique barrier, hydrophobic, and mechanical properties to cuticular layers that impact multiple biological functions of plants. These functions include resistance to biotic and abiotic stress, plant growth and development, water repellence, exchange of water and gas between plants and their environments, protection against UV radiation and retention of both polar and hydrophobic molecules [5]. All of these properties define not only the agronomical quality of crops, but also their food quality and end-uses [6]. Furthermore, it is important to take into account that different by-products concentrate cuticular layers such as cereal brans and fruit pomaces provided by the corresponding food industries. Actually, these cuticle-rich plant by-products are used as fibers for nutritional health purposes [7,8] and could be considered as a source of original lipid biomolecules for different applications as it was explored for another lipid polymer, suberin of cork tree [9,10]. Indeed, these cutin fatty acids displays interesting emulsifying and coating properties due to their amphiphilic structure [11,12]. In the last past ten years, these different settings fit with what it is now called the bioeconomy and motivated numerous researches to delineate the structure, the biosynthesis and extracellular assembly of cuticular components in relation with their (bio)functional properties. These studies have mainly profited of the use of collection of T-DNA mutants (e.g., *Arabidopsis*, rice) and other genetic tools (RNA interference, Transfer DNA, Targeting Induced Local Lesions in Genomes) coupled to the development of high resolution and complementary biophysical techniques [6]. This review will report the most recent data on the structure and assembly of the cuticular polymers with a special emphasis on the cutin polyester. 

## 2. Plant Cuticle: Evolution of an Ancestral Lipopolysaccharide Membrane?

To survive in a hostile environment and colonize the earth, plants have developed complex lipid cuticular structures that primary allow resistance to desiccation and ionizing radiations. Such complex assemblies are found only in terrestrial plants, from non-vascular bryophytes to vascular angiosperms [13]. They are not present in aquatic algae. However recent studies showed that in the transition from aquatic to land environments, *Klebsormidium flaccidum*, a semi-aquatic algae, at an intermediary stage before plant terrestrialization, displays cuticle-like structures composed by waxes and a lipid insoluble polymer [14]. However, this polymer is far from the structure of plant cutins. Indeed, it seems that lipids could form more covalent links with polysaccharides and glycoproteins than a polyester of high molecular mass. In mosses it seems that cuticles displays some characteristics of suberin due, to the presence of lipid polymer associated with phenolics [13]. Considering their fatty acid composition, some moss cutins contain dioic fatty acids [15], while others do not contain these fatty acids [16]. Finally it is interesting to look back to primitive living organisms such as photosynthetic *cyanobacteria* that can adapt to extreme environments, whose external layer is stabilized by lipopolysaccharides (LPS) [17]. Similarly, LPS are also found in the external membrane of gram-negative bacteria. These LPS of gram-negative bacteria are composed of a glycophospholipid backbone with saturated and hydroxylated fatty acid chains, esterifying a disaccharide. This backbone is linked to a polysaccharide that it is known to stimulate innate immunity in mammals [18]. In *cyanobacteria*, the LPS is not linked to a phosphate, but to a galacturonic acid, a carbohydrate also found in the pectins of plant cell walls, especially in the epidermis where cuticle is deposited [19,20,21]. Actually, covalent links between fatty acids and intra-cuticular polysaccharides have been highlighted after mild alkaline hydrolysis of the cutin polyester [22,23]. This suggests that cutin and polysaccharides could be, to some extent, covalently associated. These residual covalent links between carbohydrate and lipid polymers in cuticular layers could be considered to be reminiscent of the bacterial LPS, and a marker of the transition from aquatic to the more severe terrestrial environments. The evolution of cuticle towards a highly lipidated polysaccharide assembly is a marker of land plants and this addresses another issue on the putative specificity of structure and composition of these cutinized polysaccharides, and their roles in polyester formation and the functional properties of cuticular layers.

## 3. Reactive Chemical Groups of Cutin Monomers Determine Branching Level of Cutin

Another critical feature in cutin polyester assembly is the polymer pattern, in particular its branching level. Indeed, to form a polyester, it is necessary to have at least a hydroxyl and a carboxylate group. The building blocks of the cutin polyester are hydroxy or hydroxy/epoxy fatty acids with C16 and C18 carbon chain lengths. Glycerol and phenolics can be coupled to cutin, but are minor compounds of the cutin polymer [24]. This composition contrasts with suberin where glycerol and phenolics are abundant compounds, and where their fatty acids are mainly composed of dicarboxylic fatty acids (DCA). Supplementary mid-chain hydroxyl groups offer opportunities of branching of cutin. In tomato fruit cutin, that contains more than 80% (9/10)-16 hexadecanoic fatty acid, the secondary hydroxyl group is involved in an ester group with the carboxyl group of the same fatty acid. This was determined by transforming in situ the hydroxyl group by an ether linkage to an aromatic derivative that subsequently resisted alkaline hydrolysis, and that was easily detected by gas chromatography-mass spectrometry and Raman imaging techniques [25]. Similar conclusions were obtained by using mild alkaline hydrolysis of cutins and analysis of lipid fragments, i.e., oligomers (or oligoesters) by nuclear magnetic resonance spectroscopy [22,23]. Therefore the level of polyhydroxy fatty acids should determine the extent of branching of the cutin polymer. Indeed, linear polymers would prevail in the cutin of the pericarp of wheat kernel or in the cutin of grapefruit that contains only 8% and 15% of di- and trihydroxy fatty acids, respectively. In wheat pericarp, the major cutin monomer is 9,10-epoxy-18-hydroxyoctadecanoic acid, and in grapefruit, 10-oxo-16-hydroxyhexadecaloic acid [26]. On the contrary, high branching can be expected in tomato and pumpkin where dihydroxy fatty acids account for than 80% of the cutin fatty acids [27].

In the case of the cutin of Arabidopsis and *Brassicae* leaves and seeds, a suberin-like fatty acid composition was described [28,29,30] although the cutin of Arabidopsis flowers is mainly composed of (9/10)-16-hexadecanoic fatty acids [31]. In case of DCA-rich cutins, the polyester can be only formed if a molecule with at least two hydroxyl groups is present. The latter is provided by glycerol in suberin and it was recently shown that it is also the case for DCA-rich cutins as in Arabidopsis leaves [32]. Considering only the lipids and glycerol contents of cutin and suberin, glycerol is actually a major compound of the cutin polymer of Arabidopsis leaves, and of the suberin of potato periderm, i.e., about 30% of dry polymer mass [32,33]. On the contrary, in DCA-poor cutins, glycerol content is generally not above 5% [34]. From this glycerol contents, models of cutin assemblies were proposed for Arabidopsis [32].

Therefore, by considering the chemical diversity of cutin monomer composition in different plant species, as in different organs of the same plant, it could be expected that their extracellular assembly, i.e., polymerization, involves complex mechanisms and wide specificities.

## 4. The Monomer-to-Polymer Transition: An Interfacial and Hydrophobic Activation of Cutin Polymerization?

Numerous data are now available on the intracellular pathway of cutin monomer biosynthesis, as well as their export in the extracellular apoplastic compartment through ATP-binding cassette transporters [24,35]. Extracellular polymerization of the cutin polymer, i.e., enzymatic vs. chemical mechanisms was debated until the discovery of the cutin synthase [36,37]. The chemical mechanism of polymerization was constructed on the basis of in vitro experiments using fatty acids in solution or adsorbed on solid supports, mica, or smectite surfaces [38,39,40]. The enzymatic mechanism, i.e., existence of a cutin synthase, was demonstrated by using plant mutants, and the corresponding recombinant enzyme with its biological substrates [36,37,41]. Actually there is a range of evidence that the cutin precursor used for extracellular polymerization is a monoacylglycerol (MAG), and especially a 2-MAG, and not a free fatty acid. Indeed, specific glycerol-3-phopshate-acyltransferases (GPAT) are involved in the biosynthesis of these 2-MAG; GPAT6 and their down-regulation especially induced a significant decrease of cutin deposition [42,43]. Furthermore, down-regulation of cutin synthase leads to a slight accumulation of 2-MAG [36], suggesting that 2-MAG are used in the apoplastic compartment by cutin synthase. Besides, it was observed that, in vitro, cutin synthase could form small oligomers in an aqueous environment [36]. Actually both 2-MAG and oligomers (or oligoesters) could be substrates of cutin synthase. 

**Figure 1:**
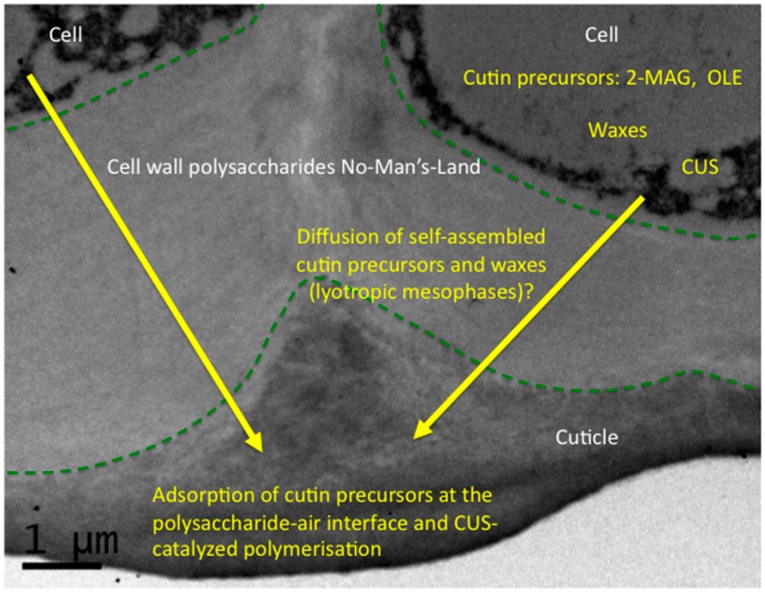
Extracellular formation of the cutin polyester in plant cuticles. Cutin monomers (2-MAG and-or OLE) are produced in the epidermal cells and are specifically polymerized within the cuticular layer. 2-MAG: 2-monoacylglycerol; OLE: cutin oligoesters; electron microscopy image of tomato exocarp at 20 days post-anthesis.

Furthermore another issue is the existence of a No-Man’s-Land of cell wall polysaccharides observed between the cutin polymer and the cells producing cutin precursors and cutin synthase (Figure 1). This means that cutin deposition does not occur immediately after the secretion of cutin precursors and cutin synthase. They have to diffuse and percolate through polar polysaccharides. Transport of monomer by proteins such as lipid transfer proteins (LTP), proteins which are abundant in the apoplastic compartment, have been suggested since a long time [44]. Indeed, LTPs play a role in the defensive responses of plants, especially loaded with lipids [45,46]. However in tomato, down-regulation of these LTPs does not seem to induce change in the cuticle [47]. A non-assisted diffusion could occur based on the peculiar self-assembly properties of cutin precursors. In this aqueous and polysaccharide-rich environment, it is important to take into account the self-assembly properties of MAG and their adsorption at air-water and oil-water interfaces as other surfactants [48]. In aqueous media MAG can form lyotropic liquid-crystalline mesophases such as cubic, reverse hexagonal, or lamellar mesophases according to the structure of the fatty acyl chain, temperature and water content [49]. The presence of polysaccharides [50] and non-polar lipids [51] can also impact self-assembly of MAG. These lyotropic structures could play a major role in facilitating lipid packing in an aqueous environments, and their further adsorption at the air-water-polysaccharide interface. Similar biomimetic assemblies combining pectin and cutin fatty acids were described [52,53]. It is also important to underline here that wax deposition is concomitant to cutin polymerization and that waxes could be considered as an oily interface. 

Interfacial activation of enzymes working at oil-water interfaces has been well described in the case of lipases [54]. However cutin synthase does not initially work at a pure oily interface, but to an interface probably enriched in amphiphilic molecules, i.e., 2-MAG of hydroxy fatty acids. An excess of amphiphilic molecules, i.e., 2-MAG, generally displaces the protein from the interface, as observed with lipases during the progression of lipolysis [55]. For some proteins, the opposite situation was observed. Indeed, the surfactant stabilizes the protein at the air–water interface [56]. Furthermore in contrast with lipases, progression of the polymerization will decrease 2-MAG content while increasing the surface of the hydrophobic interface composed by cutin polymers filled with waxes. All these questions have to be addressed in a near future to understand the formation of cutin at the air-water-polysaccharide-wax interfaces. This is especially the case for phase behavior and the interfacial properties of cutin oligomers and 2-MAG, as well as the behavior of cutin synthases at these interfaces have to be finely investigated. 

In the formation of the cutin monomers, there is another point concerning esterification of mid-chain alcohol groups of the fatty acids, especially when these groups are abundant as in tomato fruit. In general, primary alcohols are more reactive that secondary alcohol groups in the esterification reaction. However, a significant part of these secondary hydroxyl groups are actually esterified in tomato cutin [25]. Considering the work done on the esterification catalyzed by various lipases, it was shown that in absence of water, lipases are capable of esterifying both primary and secondary alcohols [57]. Furthermore some lipases are enantioselective, especially in their reverse reaction, i.e., esterification vs hydrolysis [57]. As for lipases, this enantioselectivity could favor the esterification of secondary hydroxyl groups of cutin, since the carbon atoms linked to these hydroxyl groups should normally display a specific enantiomeric configuration. By analogy with lipases, this strengthens a role of cutin synthase in the esterification of both primary and secondary alcohol groups of cutin monomers as observed in tomato cuticles [25]. To continue on this analogy, it is interesting to note that esterification of mid chain hydroxyls increases with the density of cutin deposition, e.g., in wild type vs. *cus1* mutants and during development of tomato fruit [25]. Increasing cutin density should increase its hydrophobicity to repel water molecules, a favorable condition to produce polyesters with both high polymerization mass and branching levels. Similarly, the size of cutin polyesters, estimated on the molar ratio of fatty acid to glycerol increased with cutin deposition [25].

## 5. Cutin Synthase and the Polymorphism of the GDSL-Lipase Superfamily: Relationships with the Construction of the Lipopolysaccharide Cutin Structure?

**Figure 2:**
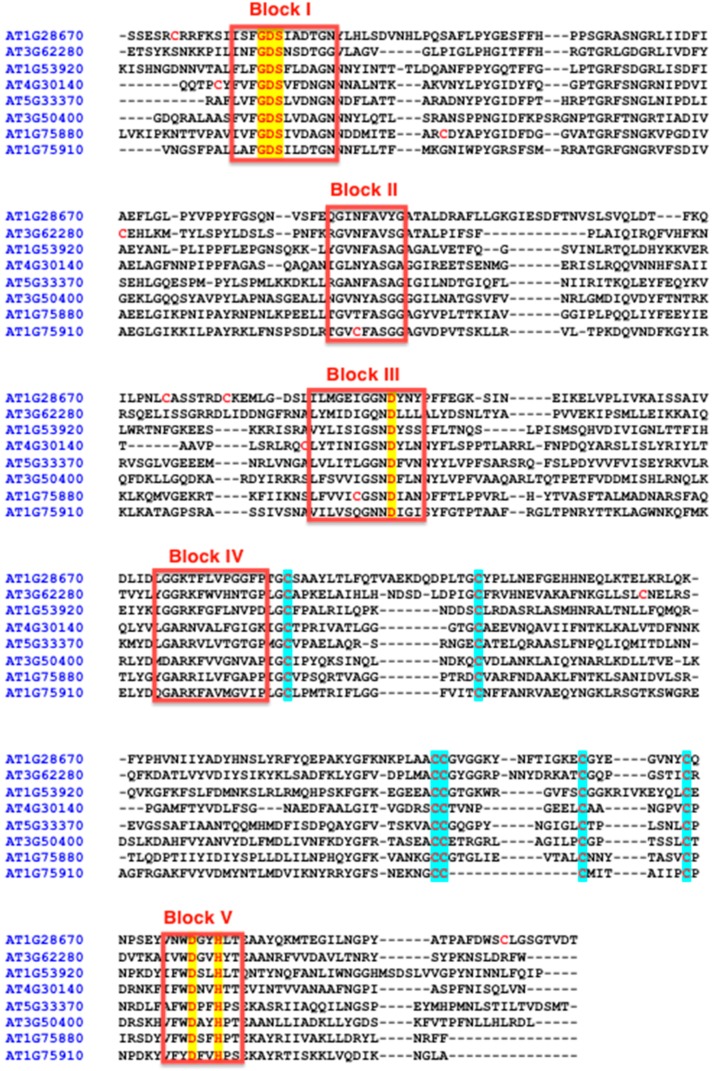
Amino acid sequences of Arabidopsis GDSL-lipases. A representative of the different clades (see last figure in the manuscript) was chosen for sequence alignment. The conserved residues involved in catalysis are highlighted in yellow while the cysteines of the six cysteine motifs (see text) are highlighted in blue. Other non-conserved cysteines are highlighted in red.

Cutin synthase belongs to a family of proteins, the GDSL lipase/esterase family that was initially described in bacteria [58]. Hundreds of genes encode these proteins in each plant species and different clades can be discriminated in regard to their amino acid sequence [59,60,61]. However, based on some consensus sequence characteristics, it is possible to limit the size of this family. Indeed we can consider first that a GDSL lipase/esterase acting in the synthesis of extracellular polyesters is synthesized through the secretory pathway, and therefore contains a signal peptide. Then, the size of the mature proteins is in the range of 320–380 amino acid residues, it contains five consensus peptide blocks as defined previously for this protein family [58], a six-cysteine motif (C-C-CC-C-C) and highly conserved residues possibly involved in a catalytic triad. The serine residue of this triad is in a conserved Gly-Asp-Ser-X located at the N-terminus, X being generally a hydrophobic residue (Leu, Val, Ile, Phe but also Asn, Thr). The strongly conserved aspartic residues located in Blocks III and V, as well as the histidine residue located in Block V can belong to the catalytic site (Figure 2). 

Homology modelling performed by using the I-TASSER (https://zhanglab.ccmb.med.umich.edu/I-TASSER/) or MODELLER protein structure prediction methods (https://modbase.compbio.ucsf.edu/modweb/) provides interesting 3D structure of these proteins. In all cases, the template used is the crystallographic structure of an autotransporter of the bacteria *Pseudomonas aeruginosa* that has a GDSL-lipase domain [62]. 

**Figure 3:**
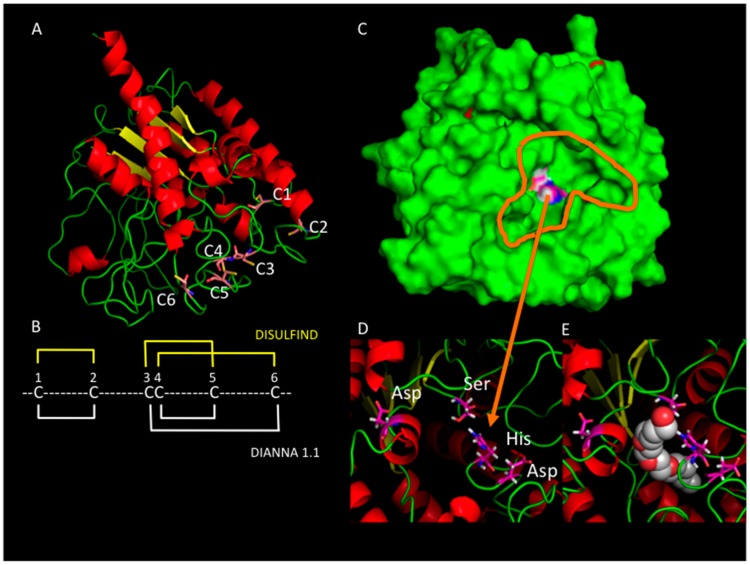
3D structure of tomato cutin synthase obtained by homology modelling and showing the catalytic triad and a putative lipid binding cavity. **A**: a 3D structure model of tomato CUS1 highlighting the cysteine motif. **B**: prediction of cysteine pairings using DIANNA 1.1 (http://clavius.bc.edu/~clotelab/DiANNA/) and DISULFIND (http://disulfind.dsi.unifi.it/) softwares. **C**: a 3D structure model of tomato CUS1 highlighting a cavity where the amino acids of a putative catalytic triad (Ser, Asp, His) are seen in blue and white. **D**: the putative catalytic triad (Ser9, Asp300, His303) and the highly conserved Asp143 residue. **E**: the catalytic site filled with a detergent molecule (C8E4) as in the template crystallographic structure of the GDSL-lipase domain of a bacterial autotransporter (pdb: 3kvn).

In these models of the tomato cutin synthase, i.e., CUS1, it is interesting to note that the conserved Asp and His residues located in Blocks V are closed to the Ser residue located in Block I (Figure 3D,E). This means that the catalytic triad could comprise Ser of Block I, and Asp and His residues of Block V, and not Asp from Block III, in agreement with another GDSL lipase with both hydrolase and acyl transferase activities [63]. Asp of the Block III could be in a calcium binding site by analogy with other lipases, where this site maintains a lid in the open state to allow the entrance of lipids in the catalytic site [64]. However, we have to be cautious with homology modeling, since the cysteines forming a conserved motif in most GDSL-lipases are not connected in the 3D models, while, in most extracellular proteins, cysteines should form disulfide bonds [65]. Indeed, cysteine pairing is predicted using different prediction methods as DIANNA [66] or DISULFIND [67] (Figure 3B). Whatever the limitations of homology modelling, it is interesting to note that some strong analogies exist between cutin synthase and lipases and, awaiting a crystal structure of a plant GDSL-lipase, such models could reasonably help us to open some exciting hypotheses on the structure-function of this family of plant proteins.

**Figure 4:**
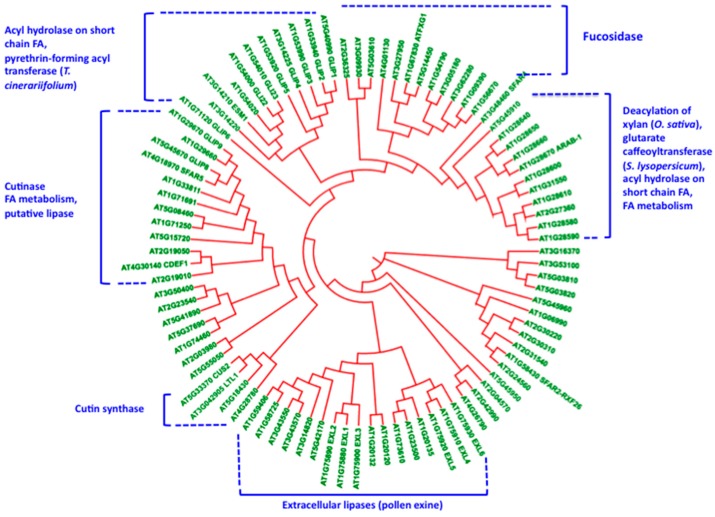
Phylogenetic tree (using the Interactive Tree of Life viewer at https://https://itol.embl.de/) of selected Arabidopsis GDSL-lipases. Clades containing proteins with functional characterization were highlighted. These functions were determined in Arabidopsis and other plant species (rice, tomato, etc.). The Arabidopsis homologs of GDSL-lipases with functional properties characterized in other plant species are presented in the phylogenetic tree.

By reducing the protein polymorphism on the criteria defined previously, different clades were highlighted that contain other GDSL-lipases (Figure 4). In some of these clades, some proteins were characterized, and their enzyme activity shown in vitro and-or in planta. Considering Arabidopsis, where we have more information on GDSL-lipase functions, it is interesting to select a specific clade for each function, e.g., for cutin synthase, for cutin hydrolase, deacetylation of polysaccharides, polysaccharide hydrolysis, fucosidase, and esterification of secondary metabolites [63,68,69,70,71,72]. Indeed, these proteins display a broad specificity of substrates associated with their multifunctional properties. Interestingly however, these proteins are either involved in the catalysis of transesterification, esterification, or hydrolysis of both hydrophilic (polysaccharides) and hydrophobic substrates (lipids and secondary metabolites such as phenylpropanoids). Regarding the structure of cuticular layers we can speculate on the role of different GDSL-lipases in the construction of this complex lipopolysaccharide composite. Furthermore the question of multiple substrates for a GDSL-lipase has to be addressed, as well as lipases that can esterify in hydrophobic environments, different type of substrates including carbohydrates [57].

## 6. Conclusions

Thanks to the use of plant models and associated genetic tools, most of the cutin biosynthesis pathway has been completed from intracellular synthesis of cutin fatty acids, extracellular transport of cutin precursors to their polymerization in the apoplastic compartment. The discovery of cutin synthase in tomato and presence of similar proteins in all land plants strengthened the role of enzymatic mechanisms in the construction of cuticular layers. However the mechanisms of action of these enzymes need to be finely explored, especially the mode of interaction with their substrates, i.e., 2-MAG and eventually oligoesters, at the polysaccharide-water-air interfaces. The organization of these substrates in a multiphasic apoplastic environment combining non-miscible hydrophobic (waxes) and hydrophilic phases, as well as liquid and solid-gel phases, also has to be determined. This could help in designing biomimetic lipopolysaccharide systems to study the mode of polymerization by cutin synthases. Indeed an interfacial activation mechanism, calcium-dependent or not calcium-dependent, induced by the adsorption of cutin synthase to its aggregated substrates could be hypothesized by analogy with lipases. Although homology modelling based on a bacterial GDSL-lipase is presently useful, determination of the three-dimensional structure of a cutin synthase will be required to move forward on these issues.

The fact that cutin synthase belongs to a large family of plant proteins that likely display similar folding but catalyze different reactions on different substrates, suggests that other members of this family could play a role in the association of polysaccharides with cutin, as well as on the rearrangement of the cutin and polysaccharides during cuticle development that occurs during the growth of plant organs. To go forward on this issue, comparison of crystallographic structures of different GDSL-lipases will be necessary, as well as having a fine description of the structure of cutinized vs. non-cutinized polysaccharides.
